# Comparative Study on Medicinal Natures (*qi*) of Black Ginseng, Red Ginseng, and Ginseng Leaves Based on Typical Deficiency-Heat Syndrome Rat Model

**DOI:** 10.1155/2022/5194987

**Published:** 2022-04-23

**Authors:** Mei-Yu Wan, Lin-Lin Liu, De-Qiang Dou

**Affiliations:** College of Pharmacy, Liaoning University of Traditional Chinese Medicine, Dalian, China

## Abstract

To elucidate the medicinal nature of black ginseng (BG) by comparison of the effects of four Chinese herbs with different medicinal natures on the deficiency-heat syndrome rat model which was established by intragastric administration of traditional Chinese drugs with hot nature, the appearance indexes, biochemical indexes, and pathological sections of thyroid and stomach were examined. In addition, the seven short-chain fatty acids (SCFAs) in rat feces were also determined by headspace gas chromatography-mass spectrometry to reveal the action mechanism of the drugs with different natures. Results indicated that all the 4 drugs could exhibit similar actions in regulating the biochemical indexes of triiodothyronine (T3), thyroxine (T4), thyrotropin-releasing hormone (TRH), thyroid-stimulating hormone (TSH), and corticosterone (CORT) representing the hypothalamus-pituitary-thyroid (HPT) and hypothalamus-pituitary-adrenal (HPA) axes of the animal. However, cold-natured cortex phellodendri (HB) and ginseng leaves (GLs) showed stronger downregulation of the AChE activity of the nervous system. Red ginseng (RG) and BG tested exhibited stronger upregulation of the liver Na^+^-K^+^-ATPase activity. Principal component analysis (PCA) showed that GLs are similar to those of HB which belongs to the cold-nature drug, whereas BG showed closer to RG which attributes to a warm-nature drug. Thus, BG could be ascribed to a warm-nature drug. Further research disclosed that RG and BG mainly regulated the acetic acid and GL and HB primarily modulated the isovaleric acid and hexanoic acid in rat feces, which could be the features of drugs with warm or cold nature on the regulation of SCFAs in rats. It is for the first time that the medicinal nature of BG and its effect on the SCFAs were examined.

## 1. Introduction

In traditional Chinese medicine (TCM), medicinal nature (called *qi* in Chinse) is a specific standard to describe the efficacy of Chinese drugs and was usually classified into four natures, i.e., cold, cool, warm, and hot natures. Generally speaking, herbs with a cold or cool nature are deemed to cure heat syndromes, such as clearing away heat, eliminating toxic substances, nourishing *yin*, and remedying hot syndromes. In contrast, herbs with a hot or warm nature are usually used to dispel cold syndromes, such as warming up the interior, supporting *yang,* and thus treating cold syndromes. Ginseng (GS), the dried roots and rhizomes of *Panax ginseng* in the family of Araliaceae, is a famous medicine and is used widely in the world. RG is the processed product of fresh ginseng, usually steamed for one time, and exhibits warm nature in accordance with China's Pharmacopoeia [1]. GLs are the dried leaves of *Panax ginseng* and exhibited cold nature, however, for the short history of application, the cold nature of GL is suspicious, and our previous experiments have not shown a positive result to confirm the cold nature of GL with hypothyroidism model [2]. BG is a newly processed product of GS by steaming and drying nine times mostly, but its nature is unclear. Previously, the chemical comparison of GS, RG, and BG was performed [3–5]. HB (called Huang-Bai in Chinese) with cold nature is the dried bark of *Phellodendron Chinense* Schneid from the family of Rutaceae and was used as a positive control drug, reflecting the therapeutic characteristics of cold nature drugs. The medicinal nature of an herb is an important index for a TCM doctor to use correctly in the clinic; it is necessary to explore the natures of GL and BG to use them correctly in the TCM clinic.

As usual, medicine with cold or cool nature is used for the treatment of diseases with warm or hot nature and vice versa. Thus, rat models established by administration with typical hot or cold herbal medicines were regarded as generally accepted methods to evaluate the nature of herbal medicine [6]. This paper deals with the exploration of the natures of BG and GL with a typical deficiency-heat syndrome rat model so as to direct their applications in TCM clinics.

## 2. Materials and Methods

### 2.1. Experimental Animals

Fifty-four specific pathogen-free (SPF) male Sprague Dawley (SD) rats (weighing 180–220 g) were purchased from the Laboratory Animal Center of Changsheng Bio-Technique Co. Ltd. (Benxi, Liaoning, China; qualification number SCXK 2020–0001). The animals were kept in an air-conditioned room (temperature, 22°C; relative humidity, 55%) and fed *ad libitum* with standard feed and water during the entire course of the study. The investigation conforms to the Guide for the Care and Use of Laboratory Animals published by the US National Institutes of Health (NIH Publication No. 85–23, revised 1996). The study protocol was reviewed and approved by the ethics regulations of the Animal Core & Welfare Committee at Liaoning University of TCM (131/2010).

### 2.2. Drugs

RG and GL were provided by Fusong County, Jilin Province (batch number: 20200910); BG was provided by Liaoning Zhongshutang Black Ginseng Co., Ltd. (batch number: 20200910); HB (dried bark of *Phellodendron chinense* Schneid. of Rutaceae) was provided by Anhui Songshantang Traditional Chinese Medicine Pieces Technology Co., Ltd. (batch number: 130506); Ginger (dried rhizome of *Zingiber officinale* Rose. of Zingiberaceae) was provided by Jilin Aodong Shihang Pharmaceutical Co., Ltd. (batch number: 21022101); *Aconitum carmichaelii* Debeaux (processed product of subroots of *Aconitum carmichaelii* Debx. of Ranunculaceae) was provided by Chengda Fangyuan Pharmaceutical Chain Co., Ltd. (batch number: 20210527); Cinnamon (dried bark of *Cinnamomum cassia* Presl of Lauraceae) was provided by Anhui Songshantang Chinese Herbal Pieces Technology Co., Ltd. (batch number: 130506). All the herbal materials were identified by Professor Dou Deqiang and Professor Wang Bing from the School of Pharmacy, Liaoning University of Traditional Chinese Medicine, and their voucher specimen were stored in the Department of Chinese Medicine of Liaoning University of Traditional Chinese Medicine.

RG, BG, GL, and HB powders were refluxed with 10 times of distilled water two times and filtered, the resulting filtrates were combined as water decoction, RG, BG, and GL were concentrated to 0.663 g/ml, and HB was concentrated to 0.884 g/ml. All the resulting extracts were stored at 4°C for later use. Model drugs were prepared as follows. A certain amount of *Aconitum carmichaelii*, Ginger, and Cinnamon were weighed according to the ratio of 1 : 1 : 1. *Aconitum carmichaelii* was decocted for 30 mins; then, Ginger and Cinnamon were added to the decoction for 20 mins, and we continue to decoct and extract it; the resulting filtrates were combined and concentrated until the crude drug contents were reached 2 g/ml and stored at −20°C.

### 2.3. Reagents and Instruments

Thyroxine (T4), triiodothyronine (T3), cyclic adenosine monophosphate (cAMP), cyclic guanosine monophosphate (cGMP), thyroid-stimulating hormone (TSH), thyrotropin-releasing hormone (TRH), corticosterone (CORT), pyruvic acid, acetylcholinesterase (AChE), protein quantification kit, and ultrafine Na^+^-K^+^-ATPase detection kit were purchased from Nanjing Jiancheng Institute of Biological Engineering (lot number 20210712). Acetic acid (CAS: 64-19-7), propionic acid (CAS: 79-09-4), butyric acid (CAS: 107-92-6), isobutyric acid (CAS: 79-31-2), valeric acid (CAS: 109-52-4), isovaleric acid (CAS: 503-74-2), caproic acid (CAS: 142-62-1), and Internal standard: 4-methylvaleric acid (CAS: 646-07-1) were all purchased from Shanghai Yuanye Biotechnology Co., Ltd.; phosphoric acid was purchased from Tianjin Damao Chemical Reagent Factory; the experimental water was ultrapure water. Other reagents were analytically pure.

Instruments used were as follows: gas chromatography-mass spectrometer type 7890B–5977 B, equipped with 7697A headspace sampler, workstation Mass Hunter Workstation B.07.00 (Agilent company, USA) and B-wax (db-1 ms) capillary column (30 *m* × 0.25 mm, 0.25 *µ*m, Agilent Company, USA); Milli-Q ultra-pure water machine (Millipore Company, USA); METTLER AE240 balance (Mettler, Switzerland); FA1004 B electronic balance (Shanghai Precision Scientific Instrument Co., Ltd.); KQ-250DB CNC ultrasonic cleaner (Kunshan Ultrasonic Instrument Co. Ltd.); TDZ4-WS low-speed centrifuge (Xiangyi centrifuge); Caretium microplate reader (Shenzhen Kaite Biomedical Electronic Technology Co., Ltd.); SHZ-82 Constant Temperature Oscillator (CHangzhou Guohua Company); UV-2100 ultraviolet spectrophotometer (Shanghai UNICO Company); DZKW-D-2 electrothermal constant temperature water bath pot (Beijing Yongguangming Medical Instrument Co., Ltd.); Micro-pipette (Gilson Company, France).

### 2.4. Animal Grouping and Administration Methods

After 7 days of adaptive feeding and a free diet, the animals were randomly divided into 6 groups, including the control group (CON), model group (MO), RG, BG, GL, and HB, with 9 animals in every group.

1.6 ml/100 g distilled water was given to the CON every morning, and 1.6 ml/100 g modeling drug was given to the other groups every morning for 14 consecutive days. From the 15th day, the CON and MO were given distilled water at 1 ml/100 g every morning, while the RG, BG, GL, and HB were given RG, BG, and GL decoction at 1 ml/100 g (crude drug 6.63 g/kg) and HB decoction at 1 ml/100 g (crude drug 8.85 g/kg) every morning, respectively. The workflow illustration of animal models and their respective drugs and doses is shown in Supplementary Materials Figure S1.

### 2.5. Determination of Appearance Indexes

After the adaptation period, the appearance indexes including the hair status, behavior activity, tongue picture, and fecal status of rats were observed every day, and a score was assayed after administration on the 7th, 14th, and 21st days (3–8 points are a mild deficiency-heat syndrome, 8–13 points are a moderate deficiency-heat syndrome, and 14 points or more are a severe deficiency-heat syndrome); scoring criteria are shown in Supplementary Materials Table S2. In addition, on the 7th, 14th, and 21st days of drug administration, the body mass of rats in every group was measured and the anal temperature and toe temperature of rats in every group were also measured by an electronic thermometer and infrared thermometer. After taking blood, the adrenal gland, liver, thymus, kidney, and stomach were dissected quickly and weighed to furnish the organ indexes of rats calculated as the weight of each organ/body mass.

### 2.6. Determination of Biochemical Indexes of the Endocrine System, Nervous System, Energy Metabolism, Substance Metabolism, and Cyclic Nucleotide System

The liver was homogenized in an ice bath with 0.9% physiological saline at a ratio of 1 : 9 and supernatant was obtained by centrifuging at 2500 r/min for 10 min and stored in a refrigerator at −80°C. The liver Na^+^-K^+^-ATPase activity was determined according to the kit instructions, and the tissue protein was determined by Coomassie brilliant blue method. Measurements of cAMP, cGMP, T3, T4, TSH, TRH, CORT, pyruvic acid contents, and AChE activity in serum were according to the operation steps of the kit manual.

### 2.7. Pathological Observation of the Thyroid and Stomach

Thyroid and stomach tissues were collected and stored in 10% formaldehyde solution, and paraffin-embedded sections were made. Pathological changes such as thyroid follicular morphology, enlargement, follicular colloid defect, follicular interstitial edema, and epithelial cell proliferation were scored under an optical microscope. Pathological changes such as inflammatory cell infiltration, gland reduction or destruction, congestion, and edema of mucosa were scored under an optical microscope. No pathological changes in thyroid and gastric mucosa were counted as 0-point, slight pathological changes as 1-point, moderate pathological changes as 2 points, and severe pathological changes as 3–4 points [7, 8].

### 2.8. Determination of SCFAs in Rat Feces

#### 2.8.1. Headspace Sampling Conditions

Headspace sampling conditions were as follows: the heating temperature of the headspace sampler sample bottle: 80°C; heating temperature of quantitative ring: 140°C; heating temperature of transmission line: 160°C; GC cycle time: 30 min; heating time: 20 min; balance time: 10 min; compression time: 0.15 min; sample injection time: 0.5 min.

#### 2.8.2. Chromatographic and Mass Spectrometry Conditions

The chromatographic column was Agilent DB-WAX(DB-1MS) capillary column (30 *m* × 0.25 mm, 0.25 *μ*m), injection mode was no diversion, the inlet temperature was 250°C, the ion source temperature was 230°C, the transmission line temperature was 250°C, and the quadrupole rod temperature was 150°C. The initial temperature of programmed temperature rise is 60°C; then heating was to 120°C at 30°C/min and to 140°C at 5°C/min for 1 min. After that, the temperature was raised to 150°C at 10°C/min for 1 min； heating was to 160°C at 5°C/min for 1 min. Finally, the temperature is raised to 230°C at 35°C/min, and the postoperation temperature was maintained at 230°C for 5 min. The carrier gas was helium, and the flow rate of the carrier gas was 1.0 ml/min.

There were electron bombardment ion source (EI), electron energy of 70ev, and solvent delay of 4.5 min, and the scanning mode was full scanning (SCAN), selective ion monitoring mode (SIM), and scanning range of 30–200 m/z.

#### 2.8.3. Determination of the SCFAs Content in Feces of Rats

Feces of rats in every group were collected at the same time point, and samples were prepared according to the test materials for pretreatment (it is shown in Section 1.1. of Supplementary Materials) and determined according to the chromatographic and mass spectrometry conditions. The response values of 7 SCFAs and corresponding internal standard compounds in every group were determined by SIM scanning mode, and their concentrations were calculated according to the daily working curve.

### 2.9. Principal Component Analysis

The SIMCA-P software was used to analyze the overall trend of the index data in every group. Taking the CON, MO, and HB as the benchmark, the distances between every drug administration group and the reference group were the main factor, and the graphic dispersion degree of each administration group was the secondary factor.

### 2.10. Statistical Methods

The experimental data were statistically analyzed by SPSS 25.0 software, and all the data were expressed as the mean ± SD. When comparing multiple groups of data, a one-way analysis of variance was used for comparison, and the tests of normality and homogeneity of variance were carried out first, which meet both normality and homogeneity of variance, and the least significant difference (LSD) test was used. The detection does not satisfy both normality and homogeneity of variance, so Dunnett's T3 test is used.

## 3. Results

### 3.1. Appearance Signs and Scores

Compared with the CON, on the 7th day of modeling, the rats in MO, RG, BG, GL, and HB began to have dry stools, hard texture of stools, yellow urine color, red tongue with little coating, red ear color, and red paw color and scored the apparent indexes (the scoring criteria are shown in Supplementary Materials Table S1), all of which showed moderate or severe deficiency-heat syndrome, indicating successful modeling (Supplementary Materials Table S2). On the 7th day after treatment, compared with the MO, the scores of RG, BG, GL, and HB decreased.

### 3.2. Effects on Body Mass and Temperature of Rats

With time, the body mass of every group showed an increasing trend. According to Supplementary Materials Table S3, on the 7th day after administration, compared with the CON, the body mass of the MO has a significant decreasing trend. Compared with the MO, the body mass of RG, BG, GL, and HB increased significantly.

Table S4 showed the changes in anal temperature in model rats with the deficiency-heat syndrome in every group in the Supplementary Materials. It can be seen from Table S4 that on the 14th day of modeling, compared with the CON, there were significant differences in anal temperature in each modeling group. After 7 days of treatment, compared with the CON, there was a significant difference in anal temperature between the MO and the BG. Compared with the MO, there were significant differences in anal temperature among the CON, GL, and HB, and the decrease in GL was more significant. According to Table S5 of Supplementary Materials, on the 14th day of modeling, compared with the CON, the toe temperature of every group increased, with a significant difference (MO:*P*=0, RG:*P*=0.001, BG:*P*=0.008, GL:*P*=0.016, HB:*P*=0). On the 7th day of treatment, compared with the MO, the toe temperature of every group decreased, and there was a significant difference between the RG and the HB.

### 3.3. Effect on the Organ Coefficient of Deficiency-Heat Syndrome Model Rats

Figure S2 showed the changes in organ coefficients of rats in every group. As compared with the CON, the elevation of adrenal gland coefficient in the MO is significantly different. Compared with the MO, the adrenal gland coefficient of other groups decreased, and there was a significant difference between CON and BG. Compared with the CON, the liver coefficient of the MO decreased. Compared with the MO, the liver coefficient of other groups increased with statistical differences, and there was a significant difference between the CON and HB. Compared with the CON, the thymus coefficient of the MO decreased, while that of other groups increased.

### 3.4. Effects on the Related Indexes of the Serum Endocrine System in Rats with Deficiency-Heat Syndrome

As shown in Table 1, the contents of T3, T4, and TRH in the MO are significantly higher than those in the CON, while those in other groups are significantly lower than those in the MO. There is a significant difference in T4 reduction in RG, while TRH content in BG is higher, while TRH content in RG, GL, and HB is lower. Compared with the CON, the contents of TSH and CORT in the MO were significantly decreased, while the contents of TSH and CORT in other groups were increased except the CON, and there was a significant difference in TSH increase in the HB. Compared with the CON, there were significant differences in CORT values of every group, and there were significant differences between the RG and GL.

### 3.5. Effects on Serum Nervous System, Energy Metabolism of the Liver Mitochondria, and Related Indexes of Serum Substance Metabolism in Deficiency-Heat Syndrome Model Rats

**Figure 1:**
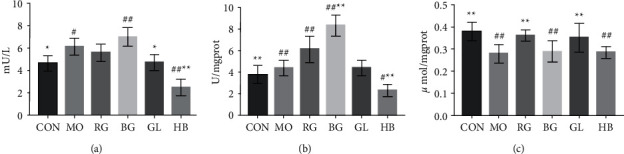
Changes in serum AChE activity in rats (a), changes in mitochondrial Na^+^-K^+^-ATPase activity in the rat liver (b), and changes in serum pyruvate content in rats (c). ^#^*P* < 0.05^, ##^*P* < 0.01 vs. CON; ^*∗*^*P* < 0.05^,^^*∗∗*^*P* < 0.01 vs. MO.

As shown in Figure 1, compared with the MO, the AChE activity of BG increased, while that of RG, GL, and HB decreased, and there was a significant difference between GL and HB, especially in HB. Compared with CON, except GL, Na^+^-K^+^-ATPase activity in other groups decreased significantly. Compared with MO, Na^+^-K^+^-ATPase activity in RG and BG decreased significantly, and there was a significant difference in the decrease of BG. While Na^+^-K^+^-ATPase activity in CON, GL, and HB increased significantly, there was a significant difference in elevation in HB. Compared with the CON, the pyruvic acid content of MO, BG, and HB decreased significantly. Compared with the MO, the pyruvate content of CON, RG, and GL increased significantly.

### 3.6. Effects on the Related Indexes of the Serum Cyclic Nucleotide System in Rats with the Deficiency-Heat Syndrome Model

As shown in Table 2, compared with the CON, the cAMP content in the MO increased, while the cAMP content in other groups decreased significantly. Compared with the CON, the cGMP content of MO, RG, and BG decreased. Compared with the MO, the cGMP content of CON, BG, GL, and HB increased, and the rising trend of CON, GL, and HB was obvious. Compared with the CON, the cAMP/cGMP ratio in the MO increased. Compared with the MO, the ratio of cAMP/cGMP in other groups decreased, and the decreasing trend was more obvious in BG, GL, and HB.

### 3.7. Changes in Pathological Morphology of the Thyroid and Stomach

**Figure 2:**
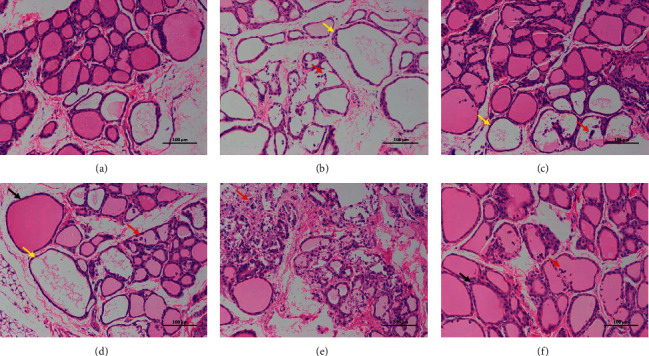
Thyroid pathological features of CON (a), MO (b), RG (c), BG (d), GL (e), and HB (f) under 200 times microscope. The black arrows indicate that the follicles are markedly swollen and vary in size, yellow arrows indicate an absence of glia in follicles, and red arrows indicate the shedding of epithelial cells.

As shown in Figure 2, the structure of thyroid follicles in the CON is clear, the morphological structure of epithelial cells is normal, and there is an abundant colloid in follicles. No obvious abnormality or inflammation is found in the stroma, and the CON corresponding score is 0. The results of MO showed that more follicles were obviously enlarged with different sizes, a small amount of colloid in follicles was missing, and a small number of epithelial cells fell off and scored 4 points. The results of RG showed that more follicles were obviously enlarged with different sizes, a small amount of colloid in follicles was missing, and a small number of epithelial cells fell off and scored 3 points. The results of BG showed that a small amount of follicular enlargement, a small amount of colloid loss, and a small amount of epithelial cell shedding were found in thyroid tissue and scored 2 points. The results of GL showed that more follicles were obviously enlarged with different sizes, more colloid in follicles was missing, local epithelial cells were necrotic and exfoliated, and nuclear pyknosis was deeply stained or broken and scored 3 points. The results of HB showed that a small amount of follicular enlargement, a small amount of colloid loss and a small amount of epithelial cell shedding were observed, and 2 points were scored.

**Figure 3:**
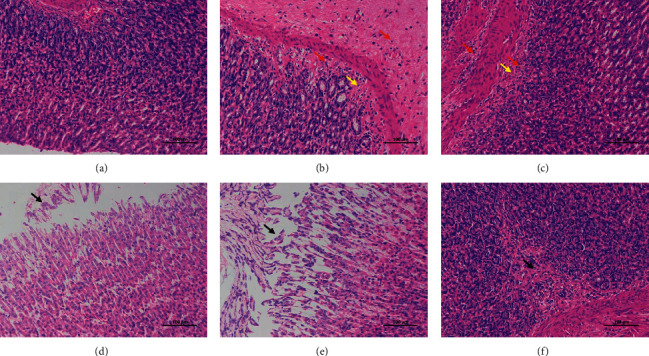
Pathological features of gastric tissue in CON (a), MO (b), RG (c), BG (d), GL (e), and HB (f) were observed under 200 times microscope. Black arrows indicate necrosis and exfoliation of epithelial cells, yellow arrows indicate the proliferation of connective tissue, and red arrows indicate punctate infiltration of lymphocytes and neutrophils in lamina propria and submucosa.

As shown in Figure 3, the results of CON showed that the structure of gastric epithelium in the gastric mucosa layer is complete, the structure of gastric pits is clear, the epithelial cells are closely arranged, the gastric glands in the lamina propria are abundant, the morphology and structure of main cells and parietal cells are normal, and there is no obvious inflammation, so the CON score was calculated as 0. The results of MO showed that a small number of epithelial cells were necrotic and exfoliated, and the nucleus was broken. A slight decrease of gastric glands and hyperplasia of connective tissue can be seen at the bottom of local lamina propria, infiltration of lymphocytes and neutrophils can be seen in lamina propria and submucosa, and MO scored 3 points. The results of RG showed that a small number of epithelial cells can be seen shedding from gastric mucosa. A slight decrease of gastric glands and hyperplasia of connective tissue can be seen at the bottom of local lamina propria, punctate infiltration of lymphocytes and neutrophils can be seen in lamina propria and submucosa, and RG scored 1 point. The results of BG showed that there were many epithelial cells shedding in the gastric mucosa. The lamina propria is rich in gastric glands, the morphology and structure of main cells and parietal cells are normal, no obvious inflammation is found, and BG scored 3 points. The results of GL showed that there were many epithelial cells shedding in gastric mucosa. The lamina propria is rich in gastric glands, the morphology and structure of main cells and parietal cells are normal, no obvious inflammation is found, and GL scored 1 point. The results of HB showed that the structure of gastric epithelium was complete, the structure of gastric pits was clear, and epithelial cells were closely arranged. A slight decrease of gastric glands and hyperplasia of connective tissue can be seen at the bottom of local lamina propria, and HB scored 1 point.

### 3.8. Determination of SCFAs

The establishment result of the SCFAs analysis method is shown in Supplementary Material Figure S3, and the result of the SCFAs methodology investigation is shown in Supplementary Material Table S6-S9. After water content correction, the determination results of 7 SCFAs in animal feces of every group are shown in Table 3.

### 3.9. Principal Component Analysis (PCA) and Variable Importance in Projection (VIP) Results of Each Index Data *In Vivo*

**Figure 4:**
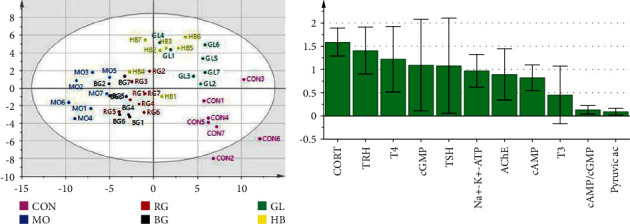
PCA analysis and VIP results of index data.

The index data (cAMP, cGMP, cAMP/cGMP, T3, T4, TRH, TSH, CORT, AChE, pyruvic acid, and Na^+^-K^+^-ATPase activity) in every group were analyzed by PCA analysis software SIMCA-P, as shown in Figure 4, and the BG is close to the RG.VIP is an important index that reflects the explanatory ability of independent variables to dependent variables. The larger the value, the stronger the explanatory ability of independent variables to dependent variables. Generally, when VIP >1, independent variables are of significant importance in explaining dependent variables [9]. The VIP of CORT, TRH, T4, cGMP, and TSH is greater than 1 by analyzing the VIP of each index, which indicates that the above indexes can better reflect the characteristics of the deficiency-heat syndrome rat model.

### 3.10. VIP of Biochemical Indexes *In Vivo* between Every Administration Group and MO

The VIP value was further analyzed by PLS-DA from the *in vivo* data between each administration group and the MO, resulting in the fact that T4, CORT, and cGMP are the indicators of RG greater than 1, T4, cGMP, CORT, and Na^+^-K^+^-ATPase are the indicators of BG greater than 1, TRH, CORT, cAMP, cGMP, and T4 are the indicators of GL greater than 1, and MO, cAMP, cGMP, T4, TRH, and CORT are the indicators of HB greater than 1, as shown in Figures S4-S7.

## 4. Discussion and Conclusions

The deficiency-heat syndrome animal model involves changes in the nerve system, endocrine system, metabolism, pathology, and other aspects [10,11], and research indicated that its principal appearance clinical symptoms are closely related to energy metabolism, which showed increasing of Na^+^-K^+^-ATPase activity, T3, T4, TRH, AChE, cGMP, and cAMP/cGMP. In addition, the apparent symptoms of rats with the deficiency-heat syndrome in this study are red claw color, ear color and tongue, restlessness, dry stool, short red urine, and sparse and erect hair, which could reflect the characteristics of the deficiency-heat syndrome [11]. Previously, the medicinal natures of *Atractylodes macrocephala* and *Poria* were evaluated successfully [12]. During the modeling period, the anal temperature of rats in all groups gradually increased as compared with the CON. Long-term administration of warm medicine could increase the anal temperature of rats, and the anal temperature decreased significantly after treatment with GL and HB. In TCM, “*Yin* and *Yang*” is the generalization of opposite attributes of interrelated things or phenomena in the universe. The *qi* of the human body is divided into *yin qi* and *yang qi* according to their different functions: *yin qi* governs cooling, moistening, tranquility, inhibition, and sinking. *Yang qi* governs warming, pus, and excitement [13]. As usual, the rising of body temperature is a feature of deficiency of *yin qi* in TCM and our result of higher body temperature in the MO is correspondent with this fact. In addition, the levels of T3 and T4 are regulated by the HPT axis, which indicates that different TCMs have different effects on thyroid hormones. The cold medicines HB and GL may play a role by reducing the contents of T3 and T4 in serum, while the modeling medicines with hot nature could increase the levels of T3 and T4. In the HPT axis, hypothalamic neurons act on pituitary neurons by secreting TRH, which promotes the secretion of TSH. TSH acts on the thyroid to maintain the normality of T3 and T4 in blood, and T3 and T4 regulate TRH and TSH by feedback, forming an HPT axis regulation loop. In this experiment, TRH increased and TSH decreased in rats with the deficiency-heat syndrome, and the hormone level was regulated by adjusting the HPT axis. Compared with the MO, TRH content in GL and HB decreased significantly, while TSH content in HB increased significantly. The deficiency-heat syndrome can make CORT negative feedback inhibit the HPA axis, which leads to atrophy and insufficiency of the adrenal cortex, and further leads to a reduction of CORT production [14]. In this experiment, the CORT content of MO decreased. Compared with the MO, the content of CORT in the RG increased significantly compared with other groups. When AChE activity was inhibited, AChE could not be effectively hydrolyzed, resulting in acetylcholine accumulation and increased nerve excitability. Compared with CON, AChE activity in MO was increased, which indicated that ACh released by cholinergic neurons was relatively increased. A large number of Ach excited gastrointestinal smooth muscle and produced strong contraction and induced contraction of gastrointestinal smooth muscle, thus promoting gastrointestinal movement. Compared with MO, the AChE activity of GL and HB was significantly decreased, indicating that the parasympathetic nerve in the body of rats with the deficiency-heat syndrome was inhibited and gastrointestinal movement was inhibited. It can be speculated that one of the important mechanisms of TCM promoting or inhibiting gastrointestinal motility effect may be achieved by increasing or reducing the distribution of cholinergic nerves in gastrointestinal intermuscular plexus and promoting or inhibiting the release of ACh [15]. Concerning the comprehensive influence on the digestive system and AChE, from the corresponding relationship with *qi* movement, GL and HB showed a downward trend, cold nature, and RG and BG showed an upward trend, warm nature, which is consistent with the final conclusion. The energy generation and heat production of the hot body are higher than those of the cold body, and the change of Na^+^-K^+^-ATPase in liver cells will affect the whole heat and cold, while the hot Chinese herbs *Aconitum carmichaelii* Debeaux and Ginger can significantly increase the ATP content in the liver. Compared with the MO, the activity of Na^+^-K^+^-ATPase in RG and BG increased significantly, while that in HB decreased significantly. According to the analysis of indexes *in vivo*, it was found that different TCMs have different ways to regulate deficiency-heat syndrome. HB and GL have stronger regulation of AChE activity, while RG and BG have stronger regulation of liver Na^+^-K^+^-ATPase activity compared with HB. By observing the pathological sections of the thyroid in rats with a deficiency-heat syndrome, it is found that the ultrastructural changes of the thyroid reflect the active function of thyroid follicular synthesis and secretion of thyroid hormone, thus increasing the energy metabolism of the body. By observing the pathological section of stomach tissue, compared with the CON, the structure of gastric mucosa in the body of the MO, the gastric glands and connective tissues at the bottom of lamina propria, and the states of lymphocytes and neutrophils in lamina propria and submucosa were significantly different. It is speculated that there will be different degrees of pathological changes in the stomach tissue, which has a certain influence on the content of SCFAs in the body. It may reflect the pathological changes in the stomach tissue and the body by adjusting the content of SCFAs. The deficiency-heat syndrome has certain effects on the thyroid and stomach of rats. RG, BG, GL, and HB have therapeutic effects on pathological changes in the thyroid and stomach of rats with the deficiency-heat syndrome.

The volatile fatty acids in feces are mainly composed of SCFAs, and SCFAs refer to organic fatty acids with 1–6 carbon chains, mainly including acetic acid, propionic acid, butyric acid, isobutyric acid, valeric acid, isovaleric acid, caproic acid, and isohexanoic acid [16], which are produced by intestinal flora metabolizing undigested carbohydrates in the intestine, and their content changes can reflect the status of intestinal flora in the body. At present, the research on SCFAs mainly focuses on maintaining HPA axis stability, anti-inflammation, immune regulation, maintaining intestinal epithelial cell integrity, and antitumor [17]. SCFAs can reduce pH value in the colon and inhibit the reproduction of pathogenic bacteria, maintain the metabolism of probiotics and the steady state of intestinal flora and nutritional colon epithelial cells, promote cell growth and metabolism, inhibit the production of proinflammatory factors, reduce the inflammatory reaction, and reduce intestinal diseases. In addition, SCFAs can inhibit the proliferation, differentiation, and metastasis of colon cancer cells [18], promote the apoptosis of cancer cells, and control the expression of protooncogenes [19]. Literature shows that Chinese medicine can play a role in treating gastrointestinal diseases by adjusting the content of SCFAs, and Chinese medicine extracts may improve the flora abundance of lactic acid bacteria and total anaerobic bacteria and increase the butyric acid content, thus achieving the efficacy of treating intestinal diseases [20]. Among them, acetic acid can not only be used as the energy substance of peripheral tissues and regulate the differentiation of adipocytes but also synthesize long-chain fatty acids, cholesterol, glutamine, *β*-hydroxybutyric acid, and other substances. Propionic acid can be used as a substrate for gluconeogenesis, which can reduce cholesterol activity and stimulate leptin growth. Butyric acid is one of the main energy substances in the colon and cecum, and isohexanoic acid is a sign of impaired intestinal microbial composition [21]. SCFAs also play an important role in maintaining the stability of the HPA axis. Acetic acid, propionic acid, and butyric acid can cross the blood-brain barrier, relieve HPA axis hyperfunction, and alleviate the behavioral changes induced by psychological stress by regulating the neuroendocrine system. SCFAs can reduce the level of adrenocorticotropic hormone-releasing hormone, but a high level (1200 mg/kg) of butyric acid can induce stress and significantly increase adrenocorticotropic hormone in plasma [22]. In this study, it was found that RG, BG, and GL could treat rats with deficiency-heat syndrome by adjusting the HPA axis and observing the pathological sections of the stomach of rats in MO; we can see that the stomach tissue of rats with the deficiency-heat syndrome will have different degrees of pathological changes, which may have a certain influence on SCFAs in their bodies. Therefore, the detection of volatile components in feces can not only reflect the content of SCFAs in the body but also reflect the state of intestinal flora in the body and then evaluate the health status of the body. Compared with the CON, the content of seven SCFAs in the experimental MO decreased, among which acetic acid, propionic acid, and butyric acid accounted for about 85%–90% of the total volatile fatty acids. The analysis of these three SCFAs showed that the RG and BG had more obvious regulation on acetic acid, and the GL and HB had stronger regulation on propionic acid and butyric acid. It reflects that the regulation effect of BG on SCFAs is similar to that of RG. The results show that the indexes of VIP >1 of BG and RG are basically the same, and the common indexes are T4, CORT, and cGMP. It shows that the two drugs have the same way to treat the deficiency-heat syndrome, which is regulated by the endocrine and cyclic nucleotide system of the body, further reflecting that their medicinal natures are close to those of RG and are warm.

According to the above results and PCA, the overall trend of each index is analyzed, and it is concluded that the medicinal natures of BG are close to those of RG and tend to be warm.

To sum up, it is for the first time that the medicinal nature of BG was evaluated to be warm nature by way of regulation of HPT (A) axes of the deficiency-heat syndrome rat model established by Chinese drugs with those natures as compared with the RG, GL, and HB, and the features of drugs with warm or cold nature on the regulation of SCFAs of rats were firstly disclosed. However, the medicinal nature of BG still needs to be examined clinically to confirm its real action on humans.

## Figures and Tables

**Table 1:** Changes of related indexes of serum endocrine system of rats (mean ± SD).

Group	T3 (ng/ml)	T4 (ng/ml)	TRH (ng/L)	TSH (m U/L)	CORT (ng/ml)
CON	9.01 ± 2.03^*∗*^	87.75 ± 10.01^*∗∗*^	180.24 ± 11.86^*∗∗*^	28.37 ± 4.01^*∗∗*^	144.40 ± 11.31^*∗∗*^
MO	12.53 ± 3.03^#^	107.26 ± 22.36^##^	195.12 ± 6.63^##^	23.27 ± 2.80^##^	93.19 ± 2.74^##^
RG	8.96 ± 4.78^*∗*^	84.30 ± 14.66^*∗∗*^	192.05 ± 9.77^#^	24.36 ± 3.63^#^	106.25 ± 3.79^##^^*∗∗*^
BG	8.85 ± 1.85^*∗*^	93.67 ± 14.34^*∗*^	199.05 ± 4.95^##^	24.35 ± 2.97^#^	103.07 ± 4.93^##^^*∗*^
GL	8.36 ± 3.05^*∗*^	93.169 ± 10.05^*∗*^	168.14 ± 6.76^#^^*∗∗*^	23.37 ± 2.89^##^	117.49 ± 9.81^#^^*∗∗*^
HB	8.20 ± 3.31^*∗*^	92.03 ± 15.04^*∗*^	181.45 ± 11.41^*∗∗*^	28.77 ± 3.76^*∗∗*^	103.16 ± 5.16^##^^*∗*^

^#^ *P* < 0.05, ^##^*P* < 0.01 vs. CON; ^*∗*^*P* < 0.05, ^*∗∗*^*P* < 0.01 vs. MO.

**Table 2:** Changes of the cyclic nucleotide system in serum of rats (mean ± SD).

Group	cAMP (nmol/L）	cGMP（nmol/L）	cAMP/cGMP
CON	59.78 ± 7.25^*∗*^	100.93 ± 5.29^*∗∗*^	0.59 ± 0.078^*∗*^
MO	65.47 ± 5.17^#^	85.25 ± 5.19^##^	0.77 ± 0.07^#^
RG	58.35 ± 4.16^*∗∗*^	89.49 ± 6.69^##^	0.65 ± 0.08
BG	54.95 ± 2.72^#^^*∗∗*^	91.64 ± 5.07^##^^*∗*^	0.60 ± 0.04^*∗∗*^
GL	53.48 ± 2.32^#^^*∗∗*^	99.47 ± 5.79^*∗∗*^	0.54 ± 0.05^*∗∗*^
HB	54.44 ± 2.11^#^^*∗∗*^	97.98 ± 3.72^*∗∗*^	0.55 ± 0.02^*∗∗*^

^#^ *P* < 0.05, ^##^*P* < 0.01 vs. CON; ^*∗*^*P* < 0.05, ^*∗∗*^*P* < 0.01 vs. MO.

**Table 3:** Determination results of 7 SCFAs in rat feces (mean ± SD).

Group	Aacetic acid (*μ*g/g)	Ppropionic acid (*μ*g/g)	Iisobutyric acid (*μ*g/g)	Bbutyric acid (*μ*g/g)	Isovaleric acid (*μ*g/g)	Pentanoic acid (*μ*g/g)	Hexanoic acid (*μ*g/g)
CON	2584.54 ± 304.16^*∗∗*^	56.06 ± 8.67^*∗∗*^	38.31 ± 2.01^*∗∗*^	515.49 ± 53.02^*∗∗*^	29.75 ± 2.28^*∗∗*^	56.59 ± 1.32^*∗∗*^	86.37 ± 21.21^*∗∗*^
MO	1451.90 ± 228.09^##^	27.15 ± 8.14^##^	18.47 ± 6.69^##^	296.86 ± 144.78^##^	11.41 ± 2.56^##^	25.48 ± 12.39^##^	8.97 ± 2.70^##^
RG	2153.04 ± 109.91^*∗∗*^	28.31 ± 5.65^##^	28.83 ± 9.67^*∗*^	337.52 ± 56.39^##^	16.61 ± 2.98^##^	28.82 ± 11.19^##^	28.59 ± 8.21^##^^*∗*^
BG	2179.64 ± 172.90^*∗∗*^	34.75 ± 3.93^##^	20.97 ± 2.95^##^	414.98 ± 117.14^*∗*^	12.08 ± 5.97^##^	31.14 ± 9.33^##^	66.63 ± 9.21^*∗∗*^
GL	1614.91 ± 240.97^##^	36.54 ± 7.16^##^	34.83 ± 16.50^*∗∗*^	439.63 ± 32.05^*∗∗*^	19.13 ± 4.60^##^^*∗*^	53.22 ± 11.78z^*∗∗*^	70.24 ± 26.25^*∗*^
HB	1752.37 ± 309.69^##^	49.81 ± 8.49^*∗∗*^	23.57 ± 2.31^##^	349.27 ± 84.40^##^	27.68 ± 10.41^*∗∗*^	34.16 ± 5.12^##^	75.52 ± 24.68^*∗*^

^#^ *P* < 0.05, ^##^*P* < 0.01 vs. CON; ^*∗*^*P* < 0.05, ^*∗∗*^*P* < 0.01 vs.MO.

## Data Availability

The data used to support the findings of this study are included within the article.

## Abbreviations

SCFAs: Short-chain fatty acids
T3: Triiodothyronine
T4: Thyroxine
TRH: Thyrotropin-releasing hormone
TSH: Thyroid-stimulating hormone
CORT: Corticosterone
HPT: Hypothalamus-pituitary-thyroid axis
HPA: Hypothalamus-pituitary-adrenal axis
CON: Control group
MO: Model group
RG: Red ginseng (group)
BG: Black ginseng (group)
GL: Ginseng leaf (group)
HB: Cortex phellodendri (group)
GS: Ginseng
PCA: Principal component analysis
TCM: Traditional Chinese Medicine
CAMP: Cyclic adenosine monophosphate
CGMP: Cyclic guanosine monophosphate
AChE: Acetylcholinesterase
ACH: Acetylcholine
EI: Electron bombardment ion source
SCAN: Full scanning mode
SIM: Selective ion monitoring mode
LSD: Least significant difference
VIP: Variable importance in projection
SPF: Specific pathogen-free
SD: Sprague Dawley.

## Supplementary Materials

Supplementary Materials The workflow illustration of animal models and their respective drugs and doses is shown in Supplementary Material Figure S1. The appearance scoring standard of rats with the deficiency-heat syndrome is shown in Table S1, and the apparent score of rats is shown in Table S2. The changes in body mass, anal temperature, and toe temperature of rats are shown in Table S3, Table S4, and Table S5, respectively. The changes in organ coefficients in each group are shown in Figure S2. The specific method of determination of SCFAs in rat feces and some results are shown in Supplementary Materials. The establishment result of the SCFAs analysis method is shown in Supplementary Material Figure S3, and the result of the SCFAs methodology investigation is shown in Supplementary Material Table S6-S9. The biochemical indexes VIP of every administration group and MO *in vivo* are shown in Figures S4-S7.
