# Supplementary material for: Comparative Study on Medicinal Natures (qi) of Black Ginseng, Red Ginseng, and Ginseng Leaves Based on Typical Deficiency-Heat Syndrome Rat Model
Source: Evid Based Complement Alternat Med. 2022 Apr 23;2022:5194987. doi: 10.1155/2022/5194987 (PMC9056217; doi:10.1155/2022/5194987)
Supplement: Supplementary Materials — The workflow illustration of animal models and their respective drugs and doses is shown in Supplementary Material Figure S1. The appearance scoring standard of rats with the deficiency-heat syndrome is shown in Table S1, and the apparent score of rats is shown in Table S2. The changes in body mass, anal temperature, and toe temperature of rats are shown in Table S3, Table S4, and Table S5, respectively. The changes in organ coefficients in each group are shown in Figure S2. The specific method of determination of SCFAs in rat feces and some results are shown in Supplementary Materials. The establishment result of the SCFAs analysis method is shown in Supplementary Material Figure S3, and the result of the SCFAs methodology investigation is shown in Supplementary Material Table S6-S9. The biochemical indexes VIP of every administration group and MO in vivo are shown in Figures S4-S7. [file 5194987.f1.docx]

**Comparative study on medicinal natures (*qi*) of black ginseng, red ginseng and ginseng leaves based on** **typical rat model of deficiency-heat syndrome supplementary material**


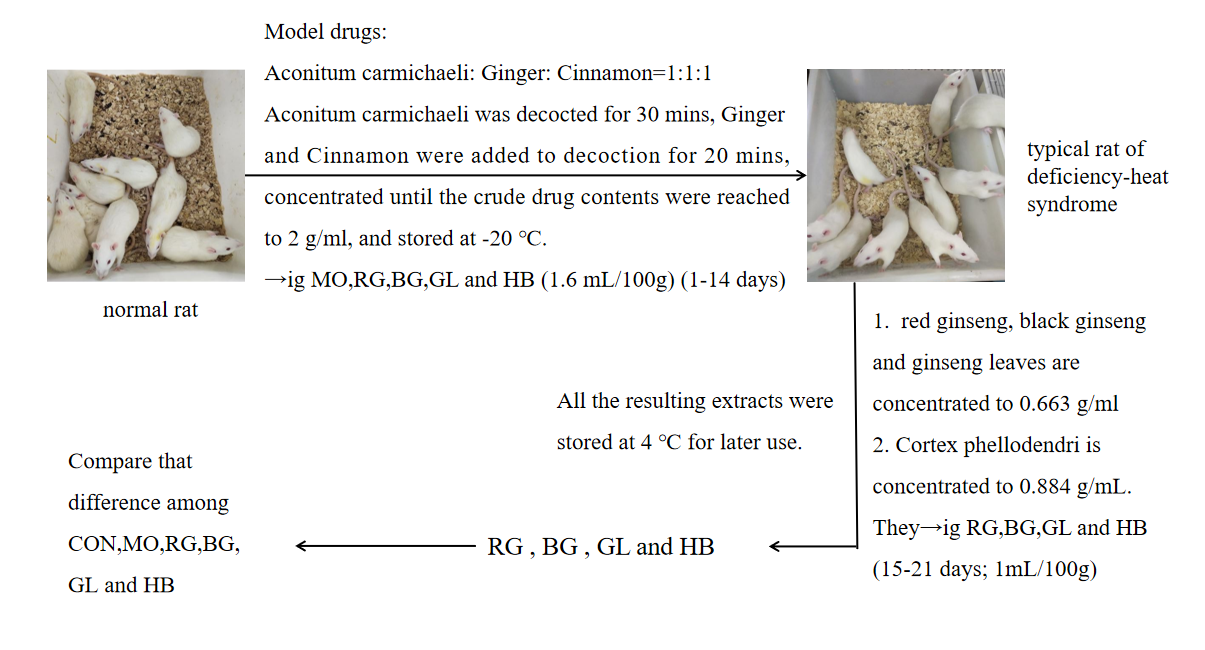


Figure S1: workflow illustration of animal models and their respective drugs and doses

Table S1: Appearance scoring standard of rats with deficiency-heat syndrome

| symptom index | 0 point | 1 point | 2 points | 3 points |
| --- | --- | --- | --- | --- |
| claw color | white | normal | partial red | redder |
| ear color | white | normal | partial red | redder |
| behavioral activity | flexibility and agility | activity increased slightly | activities have increased significantly | restless, easily surprised, afraid of people |
| fecal state | soft ellipsoid with obvious liquid on the surface | soft ellipsoid with a small amount of liquid on the surface | soft ellipsoid with no obvious liquid on the surface | dry stools, whitish stools, and reduced stools |
| urine | clear | light yellow, more quantity. | deep yellow, less quantity | short red |
| tongue quality | light red with thin white coating | light red with little coating | deep red with little coating | deep red with yellow coating |
| hair density | compact | denser | sparse | sparser |
| hair state | neat | neater | erect | upright |

Note: This table is used to evaluate the degree of deficiency-heat syndrome of rats to provide the corresponding scores. Add all the scores of symptoms to get the scores of deficiency-heat syndromes in rats, and then judge the degree of deficiency-heat syndrome.

Table S2: Apparent score of rats (mean ± SD, points)

| group | modeling 0d | modeling 14d | treatment 7d |
| --- | --- | --- | --- |
| CON | 4.44±0.882** | 5.22±1.56** | 4.77±0.66** |
| MO | 9.33±1.93## | 9.88±2.66## | 10.77±1.30## |
| RG | 8.44±1.23## | 8.66±2.66## | 9.77±2.16## |
| BG | 8.77±0.97## | 9.44±1.74## | 10.22±1.56## |
| GL | 8.22±2.90# | 9.11±1.45## | 8.77±0.83##** |
| HB | 9.44±2.12## | 8.11±1.26## | 8.77±1.09##** |

^#^*P*<0.05, ^##^*P*<0.01 vs. CON, ^*^*P*<0.05, ^**^*P*<0.01 vs.MO.

Table S3: Changes of body mass of rats (mean ± SD, g)

| group | modeling 0d | modeling 7d | modeling 14d | treatment 7d |
| --- | --- | --- | --- | --- |
| CON | 211.92±12.12 | 215.54±9.96 | 259.51±10.32 | 289.09±13.52 |
| MO | 209.93±7.54 | 221.63±19.34 | 252.36±21.92 | 266.88±22.63 |
| RG | 213.38±12.25 | 220.97±11.41 | 260.82±12.77 | 284.43±17.91 |
| BG | 207.23±8.03 | 219.82±16.48 | 258.62±15.04 | 286.69±22.40 |
| GL | 213.67±11.58 | 213.67±11.58 | 262.01±20.66 | 300.07±27.01 |
| HB | 214.01±5.74 | 220.04±8.88 | 261.15±7.14 | 276.97±12.80 |

^#^*P*<0.05, ^##^*P*<0.01 vs. CON, ^*^*P*<0.05, ^**^*P*<0.01 vs.MO.

Table S4: Changes of anal temperature in rats (mean ± SD, ℃)

| group | modeling 0d | modeling 7d | modeling 14d | treatment 7d |
| --- | --- | --- | --- | --- |
| CON | 37.06±0.46 | 37.00±0.63 | 36.57±0.29** | 36.98±0.36* |
| MO | 36.98±0.68 | 37.20±0.38 | 37.31±0.45## | 37.45±0.34# |
| RG | 37.1±0.67 | 37.08±0.56 | 37.34±0.20## | 37.16±0.42 |
| BG | 37.17±0.39 | 37.64±0.28 | 37.35±0.40## | 37.43±0.35# |
| GL | 37.31±0.48 | 37.51±0.37 | 37.43±0.49## | 36.75±0.48** |
| HB | 37.04±0.49 | 37.52±0.65 | 37.04±0.49## | 36.98±0.33* |

^#^*P*<0.05, ^##^*P*<0.01 vs. CON, ^*^*P*<0.05, ^**^*P*<0.01 vs.MO.

Table S5: Changes of toe temperature of rats (mean ± SD, ℃)

| group | modeling 0d | modeling 7d | modeling 14d | treatment 7d |
| --- | --- | --- | --- | --- |
| CON | 27.71±1.61 | 29.54±0.82 | 28.24±0.69** | 29.66±0.87* |
| MO | 27.33±0.74 | 29.36±0.57 | 30.32±0.67## | 30.53±0.97# |
| RG | 26.98±1.35 | 29.34±0.77 | 30.14±0.82## | 30.20±0.80 |
| BG | 25.97±2.39 | 29.34±0.77 | 29.85±0.86## | 29.40±0.73** |
| GL | 27.73±0.48 | 29.97±1.38 | 29.72±0.87## | 30.18±0.50 |
| HB | 28.03±1.04 | 29.91±0.70 | 30.22±0.58## | 29.67±0.38* |

^#^*P*<0.05, ^##^*P*<0.01 vs. CON, ^*^*P*<0.05, ^**^*P*<0.01 vs.MO.


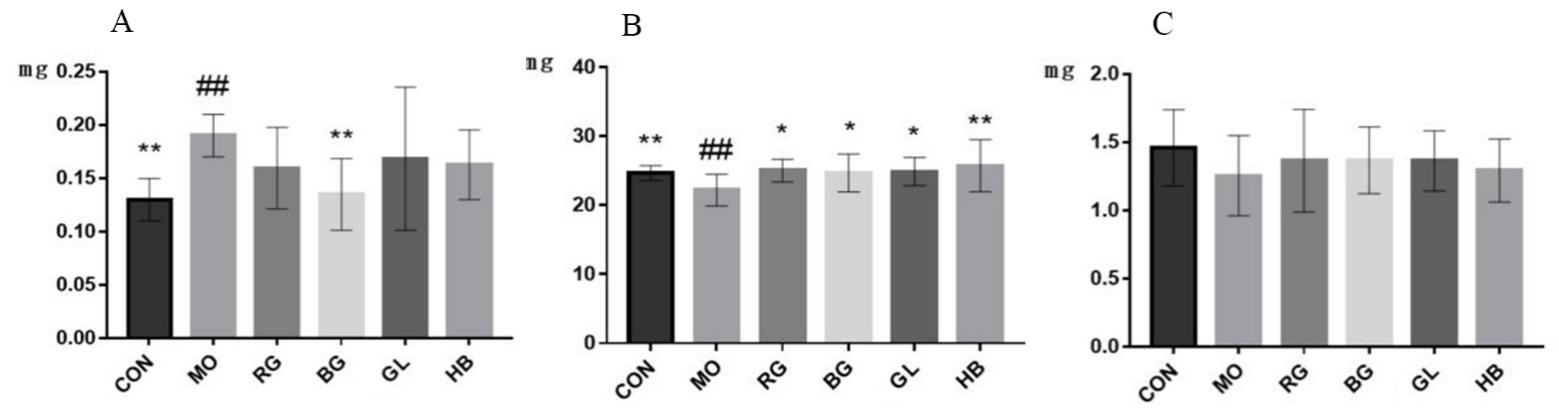


Figure S2: Changes of organ coefficients in every group. Figure S2-A shows the adrenal coefficient of rats in each group, Figure S2-B shows the liver coefficient of rats in each group, Figure S2-C shows the thymus coefficient of rats in each group. ^#^*P*<0.05, ^##^*P*<0.01 vs. CON, ^*^*P*<0.05, ^**^*P*<0.01 vs.MO.

**1. Determination of SCFAs in rat feces.**

*1.1. Preparation of test materials.* 100 mg of feces from rats in each group at the same time point was accurately weighed, and 50 μ L of 0.2% H3PO4 solution containing 4-methylvaleric acid internal standard solution (0.668 mg/mL) was added into a 20 mL headspace sampling bottle, which was quickly sealed for computer test.

*1.2. Preparation of reference substance solution.* Take appropriate amounts of acetic acid, propionic acid, butyric acid, isobutyric acid, isovaleric acid, valeric acid and caproic acid, and dissolve them with ultrapure water to prepare a reference stock solution containing 109.20 mg of acetic acid, 3.23 mg of propionic acid, 4.45 mg of isobutyric acid, 48.33 mg of butyric acid, 3.38 mg of isovaleric acid, 8.93 mg of valeric acid and 3.26 mg of caproic acid per milliliter. Take a proper amount of each reference stock solution into a 10mL volumetric flask and dilute it with water to prepare a mixed standard solution containing 10.92 mg of acetic acid, 0.323 mg of propionic acid, 0.445 mg of isobutyric acid, 0.4833 mg of butyric acid, 0.338 mg of isovaleric acid, 0.893 mg of valeric acid and 0.326 mg of caproic acid per milliliter, and dilute it to the required concentration step by step during use.

*1.3. Methodological investigation*

*1.3.1. Specificity.* Seven SCFAs standards such as acetic acid and stool samples were analyzed in full SCAN mode, and the total ion flow chromatogram was recorded. By searching NIST 14.0 library and comparing the standard samples, the qualitative identification of 7 SCFAs in samples was completed, and the attribution of chromatographic peaks was determined. According to the intensity of mass spectrum fragment peak, base peak is selected as quantitative ion of target compound, and excimer ion peak and unique fragment ion of target compound are selected as auxiliary qualitative ion. Seven kinds of SCFAs were quantitatively analyzed in the mode of selective ion detection (SIM).

*1.3.2. Linear relation.* SCFAs mixed with standard product reserve solution, purified water and diluted to 8 working concentrations. Take 10 μ L of the series of working fluids, and treat them according to the preparation method of the test sample before injection analysis. The standard curve was drawn to fit the regression equation with the standard concentration as the abscissa and the ratio of the peak area of the standard substance under different concentrations to the corresponding internal standard peak area as the ordinate.

*1.3.3. Intra-day and inter-day precision.* The feces of each group were mixed with 10mg and treated according to the preparation method of test samples. Then, the quality control sample (QC) was prepared. The intraday precision was measured by continuous injection for 6 times within one day, and the intraday precision was measured by continuous reading for 6 days. The relative standard deviation of the ratio of the peak area of SCFAs to the internal standard peak area was calculated.

*1.3.4. Stability.* To investigate the stability of fecal samples stored in a refrigerator at -80℃. Take stool quality control samples (QC) stored in the refrigerator at -80℃ for 1, 2, 4 and 7 days, and analyze the samples after processing according to the method in item 1.1. The ratio of peak area of each target SCFAs to that of internal standard was recorded, and the mass concentration of each SCFA was calculated. The stability was evaluated by the relative standard deviation of mass concentration.

The stability of SCFA solution stored at room temperature was investigated. The quality control samples (QC) were treated according to the method in item 1.1, and stored at room temperature for 0,4,8,12,24 h, and then directly injected for analysis. The ratio of peak area of each target SCFA to that of the internal standard peak was recorded. The mass concentration of each SCFA was calculated according to the working curve of the day, and the stability was evaluated by the relative standard deviation of mass concentration.

*1.3.5. Sample recovery rate.* The stool samples with known SCFA content of about 50 mg were taken and weighed accurately. According to 80%, 100% and 120% of the content of SCFA in the stool of the sample, an appropriate amount of reference product reserve solution was added, respectively. The samples were pre-treated according to the preparation method of the test product, and determined according to the chromatographic and mass spectrometry conditions. The content of each SCFA was calculated according to the recovery %=(reference substance detected-known reference substance) /reference substance added×100%, and the recovery rate and relative standard deviation were calculated.

1. **Results.**

*2.1. Establishment of SCFAs analysis method.* SCFAs are highly polar, and polyethylene glycol (PEG) modified by nitroterephthalic acid is often used as stationary phase for quantitative analysis. In this study, a variety of chromatographic columns with this kind of packing were selected, and it was found that DB-Wax column had better separation effect. After optimizing the heating program, the separation conditions of this study were determined. In addition, due to the small difference in retention time between 2-methylbutyric acid and isovaleric acid, it still cannot be separated effectively after adjusting the gradient heating program for many times. Therefore, in SIM mode, only quantitative and qualitative ion pairs of isovaleric acid were selected for analysis. At the same time, in order to reduce the system error, the internal standard method is used for quantitative analysis. According to the principles of chemical structure similar to the components to be tested, no interference, strong response and proper retention time, the internal standard was selected. After trying various volatile acid components, 4-methylvaleric acid was finally selected as the internal standard compound. See Table S6 for quantitative and qualitative ion information of 7 SCFAs and internal standard 4-methylpentanoic acid by gas chromatography-mass spectrometry. Figure S3 shows the chromatograms of the test sample before and after adding internal standard in typical blank, sample SCAN and SIM mode, as well as the typical control sample and test sample.

*2.2. Preparation method of SCFAs test solution.* Headspace analysis is a quantitative analysis based on the concentration of the substance to be measured in gas-liquid equilibrium or gas-solid-liquid equilibrium. SCFAs in feces exist in both HA (free type) and A- (ionic type), and their dissociation state is affected by the pH value of the sample. Therefore, choosing appropriate pH value to ensure that SCFAs exist as free as possible can improve the sensitivity of detection. In this study, the sensitivity of direct headspace sampling, headspace sampling with water and headspace sampling with phosphoric acid solution were investigated. The results showed that the response of SCFAs with H3PO4 solution was the highest, while the response of direct headspace sampling was the lowest. At the same time, the influence of H3PO4 addition on the detection sensitivity of SCFAs was further investigated in this study. 0,25,50,100,200 and 500μL of 2.0 %(V/V) H3PO4 was added to 100 mg samples, respectively, and then detected. The results showed that when H3PO4 solution was not added, the response value was low, and when 50μL was added, the response value reached the highest value. With the increase of H3PO4 content, the signal intensity showed a decreasing trend. Therefore, 50μL of 2.0%(V/V) H3PO4 solution was finally added.


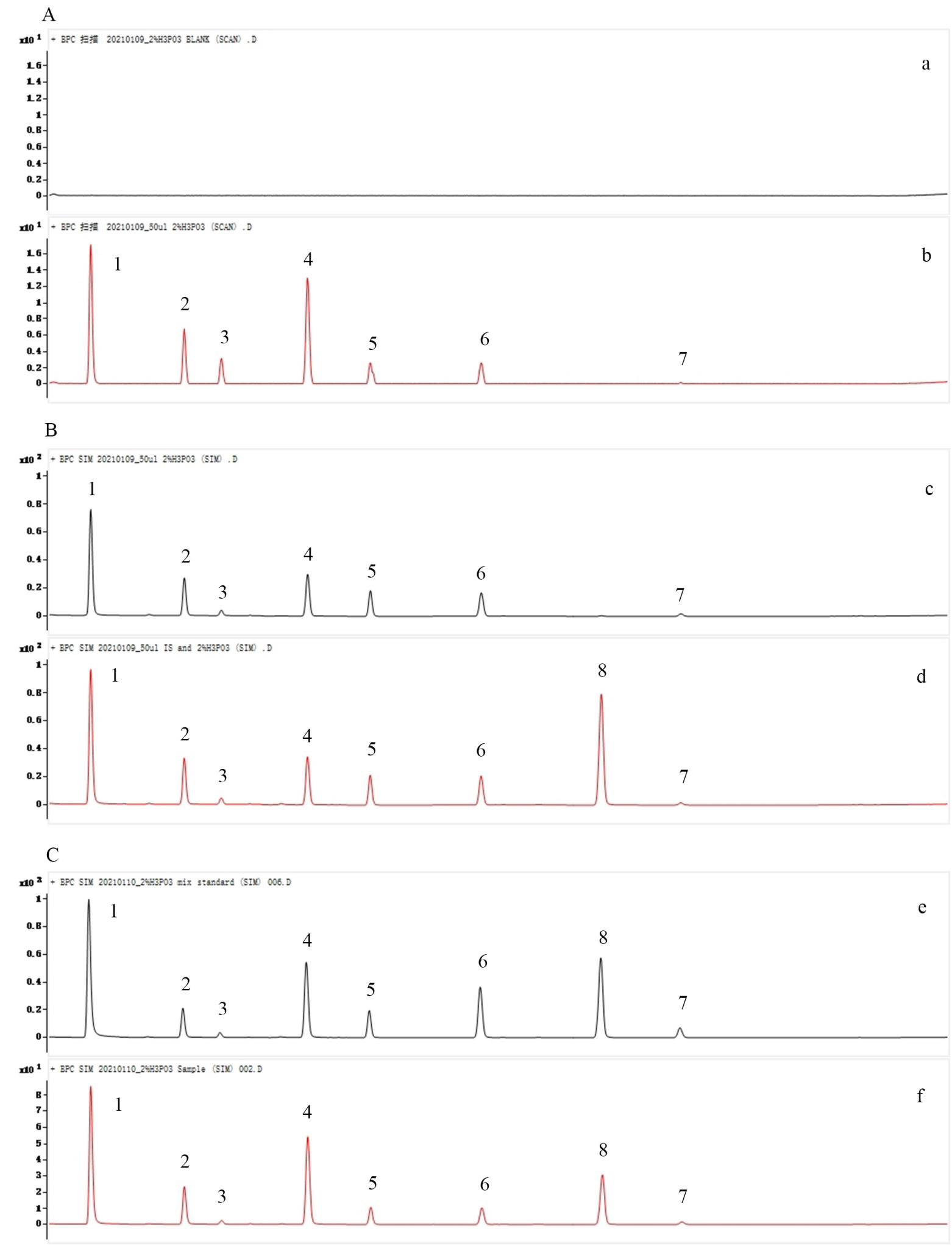


Figure S3: Typical blank and sample (SCAN) chromatogram (A), blank chromatogram (a), chromatogram of samples (b). Chromatogram of the samples before and after adding internal standard in SIM mode (B), chromatogram of the sample without internal standard (c), chromatogram of the sample with internal standard (d). Chromatogram of typical reference and test samples (c), chromatogram of reference substance(e), chromatogram of the sample (f). Acetic acid (1), propionic acid (2), butyric acid (3), isobutyric acid (4), isovaleric acid (5), valeric acid (6), caproic acid (7), and IS (8).

Table S6: Mass spectrometric parameters of 7 SCFAs and their internal standards

| serial number | compound | retention time (min) | quantitative ion (m/z) | qualitative ion (m/z) |
| --- | --- | --- | --- | --- |
| 1 | acetic acid | 4.93 | 60.0 | 43.0, 45.0 |
| 2 | propanoic acid | 5.89 | 74.0 | 45.0, 57.0 |
| 3 | isobutyric acid | 6.27 | 73.0 | 43.0, 88.0 |
| 4 | butanoic acid | 7.15 | 60.0 | 73.0, 42.0 |
| 5 | isovaleric acid | 7.81 | 60.0 | 87.0, 43.0 |
| 6 | valeric acid | 8.94 | 73.0 | 60.0, 41.0 |
| 7 | hexanoic acid | 11 | 60.0 | 73.0, 87.0 |
| 8 | 4-methylvaleric acid (IS) | 10.19 | 57.0 | 73.0, 83.0 |

*2.3.* *SCFAs methodology investigation.* As shown in Table S7, within a certain mass concentration range, the seven SCFAs have a good linear relationship between their mass concentrations and response values, and the correlation coefficients are all greater than 0.9995. Results as shown in Table S8, the RSD% of intra-day precision and inter-day precision of seven SCFAs ranged from 3.59% to 5.86% and 2.15% to 3.46%, indicating that this method has good precision. Results As shown in Table S6, the stability of all substances in feces samples stored at -80℃ in refrigerator is between 3.03% and 7.17%, which indicates that 7 SCFAs in feces have good stability within 14 days when stored at -80℃. The stability of SCFAs solution stored at room temperature for 24h is between 3.82% and 8.71%, which indicates that SCFAs solution has good stability within 24h at room temperature. Results As shown in Table S9, the recovery rate of SCFAs in stool samples ranged from 90.20% to 102.34%, and RSD% ranged from 2.48% to 5.38%, which met the relevant requirements.

Table S7: Linear regression equation, quantitative limit and detection limit of 7 SCFAs

| serial number | compound | regression equation | R | linear range (μ g/mL) | the limit of quantification (μ g/mL) | detection limit (μ g/ml) |
| --- | --- | --- | --- | --- | --- | --- |
| 1 | acetic acid | Y=0.0012X+0.0402 | 0.9996 | 36.69-2348.48 | 2.29 | 0.29 |
| 2 | propanoic acid | Y=0.0089X+0.0036 | 0.9997 | 1.01-64.52 | 0.25 | 0.06 |
| 3 | isobutyric acid | Y=0.001X+0.0013 | 0.9995 | 1.50-95.78 | 0.09 | 0.02 |
| 4 | butanoic acid | Y=0.0016X+0.011 | 0.9995 | 16.24-1039.38 | 1.02 | 0.13 |
| 5 | isovaleric acid | Y=0.0082X+0.004 | 0.9998 | 1.14-72.76 | 0.14 | 0.02 |
| 6 | valeric acid | Y=0.0063X+0.0083 | 0.9996 | 3.00-191.98 | 0.05 | 0.01 |
| 7 | hexanoic acid | Y=0.0035X+0.0047 | 0.9998 | 1.10-70.2 | 0.07 | 0.02 |

Table S8: Precision and stability of 7 SCFAs

| compound | accuracy | | stability | |
| --- | --- | --- | --- | --- |
|  | in a few days | daytime | -80℃ | room temperature |
| acetic acid | 5.08 | 2.15 | 5.31 | 5.36 |
| propanoic acid | 5.86 | 2.44 | 3.03 | 8.63 |
| isobutyric acid | 4.54 | 2.51 | 5.80 | 8.19 |
| butanoic acid | 3.78 | 3.35 | 7.17 | 7.43 |
| isovaleric acid | 3.59 | 2.44 | 6.15 | 7.88 |
| valeric acid | 3.84 | 3.46 | 6.29 | 3.82 |
| hexanoic acid | 4.75 | 2.31 | 5.46 | 8.71 |

Table S9: Recovery rates of 7 SCFAs (n=3).

| compounds | | contain (μ g) | add (μ g) | detected (μ g) | recovery (%) | RSD (%) |
| --- | --- | --- | --- | --- | --- | --- |
| acetic acid | 80% | 36.39 | 40.27 | 76.27 | 99.03 | 4.18 |
|  | 100% | 45.48 | 53.60 | 95.45 | 93.23 |  |
|  | 120% | 54.58 | 64.25 | 112.55 | 90.23 |  |
| propanoic acid | 80% | 1.24 | 1.43 | 2.60 | 95.34 | 3.05 |
|  | 100% | 1.54 | 1.65 | 3.07 | 92.65 |  |
|  | 120% | 1.85 | 2.13 | 3.91 | 96.56 |  |
| isobutyric acid | 80% | 0.71 | 0.76 | 1.50 | 103.34 | 2.61 |
|  | 100% | 0.89 | 1.10 | 1.94 | 95.98 |  |
|  | 120% | 1.06 | 1.24 | 2.22 | 93.54 |  |
| butanoic acid | 80% | 14.95 | 19.33 | 33.89 | 97.99 | 4.39 |
|  | 100% | 18.68 | 21.17 | 39.01 | 96.01 |  |
|  | 120% | 22.42 | 26.03 | 46.39 | 92.08 |  |
| isovaleric acid | 80% | 0.46 | 0.49 | 0.94 | 97.89 | 4.92 |
|  | 100% | 0.57 | 0.54 | 1.12 | 102.40 |  |
|  | 120% | 0.69 | 0.79 | 1.44 | 95.34 |  |
| valeric acid | 80% | 1.01 | 1.14 | 2.11 | 96.82 | 2.65 |
|  | 100% | 1.26 | 1.47 | 2.60 | 90.78 |  |
|  | 120% | 1.51 | 1.53 | 3.07 | 101.67 |  |
| hexanoic acid | 80% | 0.81 | 0.96 | 1.77 | 100.11 | 3.41 |
|  | 100% | 1.01 | 1.65 | 2.51 | 91.22 |  |
|  | 120% | 1.21 | 1.48 | 2.68 | 99.02 |  |

Figure S4: VIP results of index data in RG and MO

Figure S5: VIP results of index data in BG and MO

Figure S6: VIP results of index data in GL and MO

Figure S7: VIP results of index data in HB and MO
